# Evaluating the psychometric properties of the Hispanic Stress Inventory-2 Simplified version among a diverse sample of Latinos who smoke

**DOI:** 10.1371/journal.pone.0297200

**Published:** 2024-04-04

**Authors:** Ruthmarie Hernández-Torres, Francisco Cartujano-Barrera, Evelyn Arana-Chicas, Arlette Chávez-Iñiguez, Vignya Dontu, Shan Gao, Xueya Cai, Eida Castro-Figueroa, Deborah J. Ossip, Ana Paula Cupertino

**Affiliations:** 1 Department of Psychiatry and Behavioral Sciences, Memorial Sloan Kettering Cancer Center, New York, New York, United States of America; 2 Department of Public Health Sciences, University of Rochester Medical Center, Rochester, New York, United States of America; 3 Department of Medicine, Rutgers Cancer Institute of New Jersey, New Brunswick, New Jersey, United States of America; 4 Department of Biostatistics and Computational Biology, University of Rochester Medical Center, Rochester, New York, United States of America; 5 School of Behavioral and Brain Sciences, Ponce Health Sciences University, Ponce, Puerto Rico, United States of America; 6 Clinical and Translational Science Institute, University of Rochester, Rochester, New York, United States of America; 7 Department of Surgery, University of Rochester Medical Center, Rochester, New York, United States of America; The World Islamic Sciences and Education University, JORDAN

## Abstract

**Objective:**

Acculturation stress can negatively impact Latinos immigrant mental and physical health related behaviors such as smoking. It is essential to have validated and updated instruments that allow the evaluation of acculturation stress on this population. This study aims to evaluate the psychometric properties of an abbreviated version of the Hispanic Stress Inventory Version 2 (HSI2) immigration scale among Latinos who smoke.

**Methods:**

The study consisted of a secondary data analysis from a baseline assessment of Decídetexto, a mobile health (mHealth) smoking cessation randomized clinical trial. Of 457 Latinos included in the parent study, 352 immigrants who smoke were included. Construct validity was analyzed by completing a Pearson correlation coefficient matrix. Structural validity was analyzed using an Exploratory Factor Analysis (EFA). Cronbach alpha analysis was used to estimate the internal consistency of the items constituting a factor.

**Results:**

The results included an abbreviated version of the HSI2 including 52 items. From the Pearson correlation coefficient matrix with a cutoff point of 0.4, 22 of the 52 items were excluded. From the Pearson correlation coefficient matrix with a cutoff point of 0.4, 22 items were excluded. Exploratory Factor Analysis (EFA) results in six factors extracted, explaining 69.1% of the variance. According to the EFA, two items were relocated in different factors from the original scale. The HSI2 30 items scale reflected excellent reliability with a Cronbach’s alpha coefficient of 0.93. The six factors reflect acceptable to excellent reliability, ranging from 0.77–0.93 across factors. The median for the HSI2 total score was 34.00 (25–45) out of a possible total score of 150.

**Conclusion:**

Results confirmed acceptable psychometric properties of the HSI2 simplified 30-item version and provided a reliable and shorter measure of acculturation stress for Latinos groups. Having a valid and reduced measure of acculturation stress is the first step in understanding diverse ethnic groups of Latinos that are at higher risk of presenting health risk behaviors such as smoking. The present results provided the possibility of assessing the impact of acculturation stress among adults who smoke.

## Introduction

Latinos represent 18.7% of the United States (US) population (62.1 million), accounting for 44.0% of all immigrants in the US (19.8 million people) [1, 2]. Latinos, the second largest minority group in the US, include Mexicans, Puerto Ricans, Central and South Americans, Cubans, and other ethnic groups [3, 4]. For Latinos, immigration experiences include: the development of a new identity, learning a new language, and incorporating a new lifestyle and culture (e.g., clothing style, food, music, and sports) [5]. The process encompassing these unique experiences has been defined as acculturation to the US [6, 7]. The acculturation process after immigration is associated with both positive (e.g., improved quality of life) and negative experiences (e.g., discrimination, language barriers, and a sense of not belonging to the US) [8]. Acculturation stressors are frequently associated with learning a new language, changing the family dynamic, entering into new occupations, dealing with the immigration legal system, and accessing a different healthcare system [9]. In addition, acculturation stress can be associated with cultural conflict, changes in the family dynamic, and pressure to retain one’s native culture [10].

Research emphasizes that acculturation stress can negatively impact Latinos’ mental and physical health behaviors, such as sleep patterns, cigarette smoking, alcohol consumption, and diet [9, 11, 12]. For example, in a sample of 5,313 Latino adults in the US, it was found that higher levels of acculturation stress were associated with higher insomnia symptom scores [13]. Additionally, in a sample of 187 Latino immigrant men in Washington a significant association was reported between acculturation stress, discrimination, alcohol use, and alcohol-related consequences [14]. Moreover, the association between alcohol use and alcohol-related consequences was stronger for those with high levels of acculturation stress. Similarly, increased acculturative stress represents an increased smoking frequency among US Latino immigrants [15] and increased barriers to smoking cessation [16, 17].

Considering the impact of acculturation stress on the well-being of Latino immigrants, it is essential to have psychometrically sound and updated assessments that allow the evaluation of acculturation stress on this population. Four measures are psychometrically validated to measure acculturation stress among Latino populations: the Multidimensional Acculturation Stress Inventory for Adults of Mexican Origin (MASI) [18], the Acculturation Stress Inventory for Children (ASIC) [19], the Riverside Acculturation Stress Inventory (RASI), [20, 21] and the Hispanic Stress Inventory version 2 (HSI2) [22, 23]. These measures, while great, present several limitations regarding their use among the diverse Latino community. First, the acculturation stress factors evaluated are limited, and only the HSI2 considers several relevant for Latino communities, such as family-related or economic stress. Secondly, the participants are limited by country of birth, location area, and gender. For example, the HSI2 psychometric properties were tested with 575 US-born and 941 immigrant participants (*N* = 1,516). Most of the sample was from Mexico, lived in Miami, Florida, and identified as females [22].

Despite these limitations, the HSI2 continues to be widely used to evaluate acculturation stress among Latinos in the US, with extensive use in clinical and research settings [22–27]. The HSI2 was develop from the Hispanic Stress Inventory (HSI), originally published in 1990 [22]. The first HSI factor analysis resulted in two versions, an Immigrant version with 73 items comprised in five factors, and the US-born version with 59 items comprised in four factors [23]. Subsequently, in 2006, psychometric properties of an abbreviated version of the HSI (17 items) for Latino immigrants were analyzed, and the results provided initial support for the reliability and validity of the scale [26]. However, there needs to be evidence of studies using the abbreviated form in the literature. More recently, in 2016, the HSI underwent a two phases revision that resulted in a second version of the scale, the HSI2 [24]. The results included the development of two measures for HSI2; the US-born included 59 items comprised in four factors and the Latino immigrants (90 items) version with 90 items comprised in ten factors [24]. The US-born version consists of four factors: marital stress, occupational stress, parental stress, and family/culture stress. The Latin-American immigrant version is comprised of 90 items in ten factors explaining 85% of the variance: parental, occupational, and economic, marital, discrimination, immigration, marital acculturation gap, health, language, pre-migration, and family stress. The HSI2 also showed excellent reliability (Chronbach alpha = 0.97). However, the HSI2 immigrant version remains extensive (90 items), limiting its use for clinical and research purposes.

The present study aimed to evaluate the psychometric properties of an abbreviated version of the HSI2 immigration scale among Latinos from different countries of birth enrolled in “Decídetexto” [27, 28]. Decídetexto is a mobile health (mHealth) smoking cessation intervention culturally tailored for Latinos in the US who smoke. Findings from Decídetexto, the 2-arm parallel randomized controlled trial conducted with Latino adults who smoke (N = 457), showed that at six months, the self-report abstinence rate was significantly higher for the Decídetexto intervention (34.1%) compared to an education arm standard of care (20.6%) (OR = 1.99; (1.31,3.03) p = 0.01). Results from the present study will provide the opportunity to assess acculturation stress association with smoking patterns and smoking cessation outcomes.

## Materials and methods

### Study design

This study is a secondary data analysis from the baseline assessment of Decídetexto, a mobile health (mHealth) smoking cessation randomized clinical trial. It evaluates the psychometric properties of an abbreviated version (52 items) of the HSI2 among Latinos who smoke. The Hackensack University Medical Center, the University of Rochester Medical Center, and The University of Kansas Medical Center Institutional Review Boards approved and monitored the study procedures. A Community Advisory Board informed the study design and implementation [27]. Informed consent was signed electronically by subjects on REDCap via a study iPad before enrolling in the study. The abbreviated HSI2 consisted of 52 items that were distributed in ten factors as proposed in the original scale: Parental Stress, Occupation and Economic Stress, Marital Stress, Discrimination Stress, Immigration-Related Stress, Marital Acculturation Gap Stress, Health Stress, Language Related Stress, Pre-Migration Stress, and Family-Related Stress. The details of the clinical trial intervention and protocol have been reported in previous articles [27–29].

### Participants

A total of 457 US Latinos from 21 Latin American countries who smoke were enrolled in the parent study. Participants were recruited from August 2018 through March 2021 in four states (New Jersey, Kansas, Missouri, and New York) by multicultural and bilingual recruiters (English and Spanish). The recruitment strategies approaches were 1) mass recruitment strategies, 2) direct, high-effort strategies, and 3) direct, low-effort strategies [29]. Inclusion criteria included: 1) self-identify as Hispanic or Latino, 2) know how to read and speak English and/or Spanish, 3) are at least 21 years old, 4) have smoked cigarettes for at least six months, 5) smoke cigarettes three or more days within a typical week, 6) report interest in quitting smoking in the next 30 days, 7) have an active cellphone with unlimited text messaging capability, 8) know how to send and read text messages, and 9) be willing to complete two study visits (at the beginning and six months) and at least one phone call between the visits (to complete the Week 12 assessment). Exclusion criteria included: 1) use of other tobacco products more than one day within a typical week, 2) current participation in any other smoking cessation program or use of any type of medication to quit smoking, 3) having a household member participating in the study, 4) being pregnant, breast-feeding, or planning to become pregnant in the next year, and 5) planning to move out of a current residential address in the upcoming six months. Detailed recruitment strategies, eligibility, and support data have been previously reported [27–29]. The baseline assessment was completed in person by trained bilingual research staff. Participants were able to complete the assessments in either Spanish or English. For the present study, 105 participants who identified the US as their country of birth were excluded, resulting in a final sample of 352 Latino immigrants who smoke.

### Measures

#### Sociodemographic information

Sociodemographic characteristics included age, gender, country of birth, generation in the US, primary language (English or Spanish), years living in the US, education level, marital status, employment status, and annual household income. In addition, years living in the US were categorized as <10, 10 to 19, and ≥ 20.

#### Hispanic stress inventory-2 (HSI2)

An abbreviated version of the HSI2 was developed to minimize survey fatigue. The Community Advisory Board (CAB) and the study team reviewed the 90 HSI2 immigrant version survey items. Using a consensus approach, the CAB and study team selected the items that best contributed to understanding the types and nature of stressors experienced by the Latino community. Moreover, items were discussed collaboratively in the domains of clarity and comprehension. A total of 52 items were included in the final questionnaire [S1 Data]. The items were distributed in ten factors as proposed in the original scale: Parental Stress (3), Occupation and Economic Stress (12), Marital Stress (3), Discrimination Stress (10), Immigration-Related Stress (9), Marital Acculturation Gap Stress (3), Health Stress (3), Language Related Stress (3), Pre-Migration Stress (3) and Family-Related Stress (3). Each item consisted of a sentence describing experiences according to the mentioned factors. First, participants indicated if they had experienced the stressor (Yes or No). If they answered yes, or experienced the stressor, they rated the magnitude of the stressor using a 5-point Likert-type scale ranging from 1 = Not at all worried/tense to 5 = Extremely worried /anxious. As the authors of the scale suggested [24], for items where participants reported they had not experienced a stressor, the appraisal score was coded to 1 (not at all worried/ tense). Items classified as non-applicable were scored with 0. Total scores range from 0 to 150, with higher scores reflecting higher acculturation stress levels.

### Analysis

Data were analyzed using SPSS (version 28.0.1.0) with a 5% significance level (p = 0.05). Since the HSI2 used in the present study was an immigrant version, Exploratory Factor Analysis (EFA) was performed for immigrant participants (n = 352). The EFA approach is a variable reduction technique to identify latent clusters and underlying factor structures of a set of variables [30, 31]. Variable distributions were assessed with the Shapiro-Wilk test to determine the statistical approach required. However, scores were not normally distributed (*p* < .01) for any variables; thus, we used non-parametric methods to describe the outcomes (e.g., median and interquartile ranks). Listwise deletion was used to remove all data for a case with one or more missing values considering the percentage of missing data [32].

Psychometric analysis began by constructing a Pearson correlation coefficient matrix, resulting in the retention of 30 out of 52 items significantly correlated with more than 95% of the other variables, with a coefficient cut-off point of 0.4. Next, the Kaiser-Meyer-Olkin test was conducted to evaluate the sample adequacy for the EFA. The EFA approach used principal component extraction with orthogonal varimax rotation for the factor analysis on the HSI2 30 items. We opted for an exploratory factor analysis due to the need for more previous exploration of the abbreviated measure. We considered item loadings, internal consistency, and item content to delete items. Items with factor loadings less than 0.6 were removed [30, 31]. Statistics for the EFA are based on cases with no missing values for any variable used. After accepting a final EFA model, we calculated factor scores by averaging the items indicated to load on the factors that could be interpreted. These generated factor scores were used in subsequent analyses. Cronbach alpha analysis and Split-half reliability were used to estimate the instrument’s internal consistency.

## Results

Participants’ characteristics and sociodemographic data are summarized in Table 1. The sample included 194 men (55.1%) and 158 women (44.9%). The mean age was 49.9 years old (SD = 10.8), 67.3% had a high school education or higher, and most participants were married (55.7%) and employed (65.3%). The majority of participants immigrate from Mexico (17.1%), Puerto Rico (12.7%), Dominican Republic (11.6%), and Colombia (10.5%), Cuba (6.3%), Perú (1.1) and El Salvador (1.5). Most participants had lived in the US for over 20 years (60.8%), and 87.5% preferred to complete the intervention in Spanish. Missing data for the HSI2 varied from .2% to 10.2% resulting in 36 participants excluded from the EFA. No more than one participant refused to answer each item (.2%).

**Table 1:** Sociodemographic characteristics of the study participants (n = 352).

Characteristic	Statistics	
Age	Mean	SD
	49.2	10.8
**Gender**	n	%
Man	194	55.1
Woman	158	44.9
**Country of Birth**		
Mexico	78	17.1
Puerto Rico	58	12.7
Dominican Republic	53	11.6
Ecuador	34	7.4
Colombia	48	10.5
Cuba	29	6.3
Perú	5	1.1
El Salvador	7	1.5
Other	40	8.8
**Preferred Language**		
Spanish	308	87.5
English	44	12.5
**Years living in the US**		
< 10	59	16.8
10 to 19	79	22.4
> 20	214	60.8
**Education level**		
< High school	115	32.7
>High school	237	67.3
**Marital Status** *		
Married/cohabiting	196	55.7
Not married	154	44.3
**Employment Status**		
Employed	230	65.3
Unemployed	122	36.7
**Annual Income** *		
< $30,000	139	44.4
$30,000-$49,999	78	24.9
>$50,000	96	30.7
**Children < 18**	135	38.2
**Household Number**		
1 to 2	318	90.3
3 or +	34	9.7

SD: Standard deviation

*n values will not always sum 352 due to small amounts of missing data.

### Construct validity

From the data pre-processing step using the Pearson correlation coefficient matrix with a cutoff point of 0.4, 22 items were excluded. Kaiser-Meyer-Olkin’s measure of sampling adequacy was 0.89, confirming the factorability of the correlation matrix of the HSI2 30-item version. Bartlett’s Test of Sphericity indicated that variables were correlated (χ^2^  =   6122.36, d.f.  =  435, p< .000). Following the rule for extracting factors (eigenvalue greater than 1), six factors were extracted, explaining 69.1% of the variance in the six domains. These factors were 1) Occupation and Economic Stress, 2) Discrimination Stress, 3)Immigration-Related Stress, 4) Marital Acculturation Gap Stress, 5) Language Related Stress, and 6) Pre- Migration Stress. Factor loadings with item reduction of the short version of HSI2 using Principal Component Extraction analysis with varimax rotation are presented in Table 2. Item 10 (10: There was a lack of respect in our marital relationship) was initially included in the Marital Stress domain. However, Exploratory Factor Analysis (EFA) results show a relocation of item 16 to the Marital Acculturation Gap Stress factor with a factor load of 0.758, eliminating the Marital Stress factor. Similarly, item 15 from the Discrimination Stress (15. Because I am Hispanic/Latino, I was given the lowest position at work) was relocated with factor loadings of 0.62 to Occupational and Economic Stress.

**Table 2:** Factor loadings of the short version of hispanic stress inventory– 2 using principal component extraction with varimax rotation.

Item	*Factors*					
	*1*	*2*	*3*	*4*	*5*	*6*
	Immigration Related Stress	Occupation and Economic Stress	Discrimination Stress	Marital Acculturation Gap Stress	Language-Related Stress	Pre-Migration Stress
1. Because I am Hispanic/Latino, it has been hard to get promotion.		0.77				
2. Because I am Hispanic/Latino, I have been expected to work hard.		0.75				
3. Because I am Hispanic/Latino, I have been paid less than others.		0.77				
4. I have felt that due to work, the rhythm of my life has changed.		0.76				
5. I have been forced to accept low paying jobs.		0.73				
6. Because of the importance of getting ahead in my job, I had to compete with others.		0.73				
7. I did not get the job I wanted because I did not have the proper skills.		0.69				
8. I have been criticized about my work.		0.66				
9. Others have been too worried about the amount and quality of work I do.		0.70				
10. There was a lack of respect in our marital relationship.				0.76		
11. I have been discriminated against.			0.76			
12. I have felt unaccepted by others due to my Hispanic culture.			0.82			
13. I was treated "less than" other Americans because I am Hispanic/Latino.			0.83			
14. I experienced discrimination because of the color of my skin.			0.80			
15. Because I am Hispanic/Latino, I was given the lowest position at work.		0.62				
16. My legal status has been a problem in getting a good job.	0.78					
17. I feared the consequences of deportation.	0.77					
18. I have thought that if I went to a social or government agency I would be deported.	0.84					
19. Because I did not have a SSN, I could not apply for or find good employment.	0.86					
20. My legal status has limited my contact with family and friends.	0.81					
21. I have been questioned about my legal status.	0.74					
22. Since I did not have legal documentation, I was overworked at my job.	0.77					
23. Because of the lack of legal documentation, I could not get quality health care.	0.78					
24. Because I did not have a driver’s license, it was difficult to get to and from work.	0.68					
25. It has been difficult for me to understand why my spouse wishes to be more Americanized.				0.85		
26. It has been difficult for my spouse and me to combine Hispanic/Latino and American culture.				0.84		
27. Because of language barriers, I could not communicate with others.					0.81	
28. Because I do not know enough English, it has been difficult for me to interact with others.					0.84	
29. My family was exposed to poverty in my home country.						0.80
30. I was forced to leave my home country due to poverty.						0.83

### Internal consistency reliability

Cronbach’s alpha coefficient was 0.93 for the 30-item scale. The six factors reflect acceptable to excellent reliability, ranging from 0.77–0.93 across factors (Table 3) [33]. The split-half reliability coefficient in the total scale was 0.68, indicating satisfactory internal consistency. The median for the HSI2 total score was 34.00 (25–45) out of a possible total score of 150. The median and interquartile range for the six factors scores are included in Table 3.

**Table 3:** Coefficient cronbach’s alphas, median, and interquartile range for scores on the hispanic stress inventory—2 simplified version factors.

Factors	Cronbach’s Alpha	Items	Median (Interquartile range: Q1-Q3)
1. Occupation and Economic Stress	0.92	10	11.0 (10.0–17.0)
2. Discrimination Stress	0.76	4	3.0 (4.0–5.0)
3. Immigration-Related Stress	0.93	9	9.0 (6.0–9.0)
4. Marital Acculturation Gap Stress	0.77	3	3.0 (0–3.0)
5. Language Related Stress	0.83	2	2.0 (2.0–5.0)
6. Pre-Migration Stress	0.84	2	2.0 (2.0–2.0)
**HSI2 Total score**	0.93	30	34.0 (25.0–45.0)

## Discussion

The current study assessed the psychometric properties of a shortened version of the Hispanic Stress Inventory (HSI2) for Latinos who smoke from diverse ethnicities and backgrounds. The psychometric analyses supported a shortened 30-item version of the HSI2. The Kaiser-Meyer-Olkin test results reflected an adequate sample size for the HSI2 30 items version. Furthermore, Bartlett’s Test of Sphericity indicated that the HSI2 short version demonstrated good construct validity. Exploratory Factor Analysis (EFA) showed that the HSI2 short version (30 items) supported the factorial structure of the instrument, similar to analyses presented in the 90-item version [34]. Six of the ten factors included in the original version were maintained. The factors are 1) Occupation and Economic Stress, 2) Discrimination Stress, 3)Immigration-Related Stress, 4) Marital Acculturation Gap Stress, 5) Language Related Stress, and 6) Pre- Migration Stress. The scale showed α values with acceptable-excellent reliability and satisfactory internal consistency. These findings suggest that the HSI2 short version appropriately fits the theoretical conceptualization and was established when the scale was developed [22, 24] and supports the validity of this instrument.

A significant contribution of the present study is the inclusion of a diverse sample of Latino immigrants from different countries of birth and areas of residence. The participants in the study immigrate from eight different countries in Latin America, including Colombia, Cuba, Dominican Republic, Ecuador, Mexico, Puerto Rico, Peru, and El Salvador. In addition, the participants resided in four central and northeast states of the United States (Kansas, Missouri, New Jersey, and New York). The HSI2 short version could be applied to Latino immigrants from diverse countries of birth. The psychometric validation of the HSI2 short version addresses one of the limitations of previous studies validating acculturation stress measures where the samples were homogeneous regarding country of birth and residential area [34]. Also, the present findings highlight the need to continue updating acculturation stress measures among Latinos and other ethnic minority groups impacted by immigration processes. Additionally, evaluating the psychometric properties of the HSI2 among Latinos who smoke contributes to the validity and reliability of the instrument in populations presenting documented risk behaviors associated with acculturative stress, such as smoking [15, 16]. This will allow the assessment of the association between acculturative stress and smoking behavior and cessation with greater precision.

The HSI2 short version is a valuable multifactorial measure of acculturation stress relevant to Latino immigrant communities. Our results highlight and confirm the role of language, work, the social environment, and discrimination as psychosocial aspects of immigrant Latinos today [14, 35, 36]. Therefore, acculturation stress should be addressed as a multifactorial construct in minorities impacted by the adaptation processes to a new culture. However, it is essential to mention that the analysis eliminated four factors included in the last HSI2 version: parental, family, marital, and health stress. The exclusion of these factors does not mean that Latinos who smoke included in the study do not suffer from parental, health, family, or marital stress. For example, parental stress is documented as having a significant association with psychological distress and greater substance use, including cigarette and other tobacco and alcohol use among Latinos [37]. In addition, in Latino immigrants, family-cultural conflict and pressure to retain one’s native culture were identified as risk factors for acculturation stress [38]. Loss of cohesion in the family is thought to negatively impact the ability of the family members to cope with other acculturation stressors [39]. Similarly, related to health, Latinos experience more barriers to healthcare services (insurance coverage, cost of care, and usual source of care) compared with other racial and ethnic groups in the US [40].

The present study has several limitations regarding methodological procedures. A Community Advisory Board (CAB) and research team selected the 52 items in the study. Still, no standardized process existed, resulting in the exclusion of 38 items from the original 90 items scale, with ten items eliminated in the parental stress factor, nine in the marital stress factor, five in the health-related stress factor, and two in the family-related stress factor. Even when we used a CAB and multidisciplinary research experts to choose the items, including other selection criteria, such as a scoring checklist, evaluating the items included and excluded would be more accurate. Secondly, excluding the four factors could be explained by protective factors in our sample previously identified in the literature, such as years living in the US and marriage. In our study, most of the sample have lived in the US for over 20 years and were married. However, we did not perform statistical analysis to confirm the presence of protective factors among Latinos who smoke. Future studies should include the entire set of the four eliminated factors.

Another significant limitation is that the study was conducted with Latino immigrant adults who smoke, which may limit generalizability to the Latino immigrant and US-born population in the United States. Considering the significant growth of Latinos in the United States during the last decade and its diversity, future analysis should compare acculturation stress levels by country of birth. Finally, though internal consistency was good, we only included baseline data and the HSI-2 as a measure of stress. This limited the psychometric analysis regarding test-retest reliability and criterion validity analysis. Future studies should consist of repeated assessments of the HSI2 among participants to evaluate its reliability. In addition, future studies should assess the validity by running criterion validity analysis and some correlations with other constructs (e.g., perceived stress).

## Conclusion

Acculturation stress measures are needed to evaluate the impact of immigration and acculturation processes among Latinos in the US. Results confirm acceptable psychometric properties of the HSI2 simplified 30-item version and provide a reliable and shorter measure of acculturation stress for Latino immigrant groups. Having a valid and reduced measure of acculturation stress is the first step in understanding diverse ethnic groups of Latinos. Also, the present results provide the opportunity to assess acculturation stress’s impact on health behaviors such as smoking. This can guide the development of culturally responsive adaptations of interventions to promote health equity in this large population.

## Supporting information

S1 Data (DOCX)
