# Supplementary material for: Evaluating the psychometric properties of the Hispanic Stress Inventory-2 Simplified version among a diverse sample of Latinos who smoke
Source: PLoS One. 2024 Apr 4;19(4):e0297200. doi: 10.1371/journal.pone.0297200 (PMC10994365; doi:10.1371/journal.pone.0297200)
Supplement: S1 Data — (DOCX) [file pone.0297200.s001.docx]

**Hispanic Stress Inventory**

**52 items Version**

| Has this occurred to you in the last 12 months? | ***Yes, I feel…*** | | | | | **No** |
| --- | --- | --- | --- | --- | --- | --- |
|  | **Not at all worried**  **/tense** | **A little worried**  **/tense** | **Moderately worried**  **/tense** | **Very worried**  **/tense** | **Extremely worried**  **/tense** |  |
| 1. My children have not respected my authority the way they should. |  |  |  |  |  |  |
| 1. I have thought that my children used illegal drugs. |  |  |  |  |  |  |
| 1. My children have been influenced by bad friends. |  |  |  |  |  |  |
| 1. Because I am Hispanic/Latino, it has been hard to get promotion. |  |  |  |  |  |  |
| 1. Because I am Hispanic/Latino, I have been expected to work hard. |  |  |  |  |  |  |
| 1. Because I am Hispanic/Latino, I have been paid less than others. |  |  |  |  |  |  |
| 1. I have felt that due to work, the rhythm of my life has changed. |  |  |  |  |  |  |
| 1. I have been forced to accept low paying jobs. |  |  |  |  |  |  |
| 1. I have put pressure on myself to provide more things for my family. |  |  |  |  |  |  |
| 1. Because of the importance of getting ahead in my job, I had to compete with others. |  |  |  |  |  |  |
| 1. I did not get the job I wanted because I did not have the proper skills. |  |  |  |  |  |  |
| 1. Both my spouse and I have had to work. |  |  |  |  |  |  |
| 1. I have been criticized about my work. |  |  |  |  |  |  |
| 1. The pressures to achieve economic success have made me stop going to church. |  |  |  |  |  |  |
| 1. Others have been too worried about the amount and quality of work I do. |  |  |  |  |  |  |
| 1. There was a lack of respect in our marital relationship. |  |  |  |  |  |  |
| 1. My spouse and I talked about divorce. |  |  |  |  |  |  |
| 1. My spouse acted too *Machista*. |  |  |  |  |  |  |
| 1. I was called names and treated badly because I am an immigrant. |  |  |  |  |  |  |
| 1. Because I am Hispanic/Latino, I was treated like a slave. |  |  |  |  |  |  |
| 1. I have been discriminated against. |  |  |  |  |  |  |
| 1. I have felt unaccepted by others due to my Hispanic culture. |  |  |  |  |  |  |
| 1. I was treated "less than" other Americans because I am Hispanic/Latino. |  |  |  |  |  |  |
| 1. I experienced discrimination because of the color of my skin. |  |  |  |  |  |  |
| 1. Because I am Hispanic/Latino, I was given the lowest position at work. |  |  |  |  |  |  |
| 1. Members of my family have experienced discrimination. |  |  |  |  |  |  |
| 1. Because of my poor English, people treat me badly. |  |  |  |  |  |  |
| 1. I have seen friends treated badly because they are Hispanic/Latinos. |  |  |  |  |  |  |
| 1. My legal status has been a problem in getting a good job. |  |  |  |  |  |  |
| 1. I feared the consequences of deportation. |  |  |  |  |  |  |
| 1. I have thought that if I went to a social or government agency I would be deported. |  |  |  |  |  |  |
| 1. Because I did not have a SSN, I could not apply for or find good employment. |  |  |  |  |  |  |
| 1. My legal status has limited my contact with family and friends. |  |  |  |  |  |  |
| 1. I have been questioned about my legal status. |  |  |  |  |  |  |
| 1. Since I did not have legal documentation, I was overworked at my job. |  |  |  |  |  |  |
| 1. Because of the lack of legal documentation, I could not get quality health care. |  |  |  |  |  |  |
| 1. Because I did not have a driver’s license, it was difficult to get to and from work. |  |  |  |  |  |  |
| 1. My spouse and I disagreed about going back to our home country. |  |  |  |  |  |  |
| 1. It has been difficult for me to understand why my spouse wishes to be more Americanized. |  |  |  |  |  |  |
| 1. It has been difficult for my spouse and me to combine Hispanic/Latino and American culture. |  |  |  |  |  |  |
| 1. I did not have health insurance to cover my illness. |  |  |  |  |  |  |
| 1. I had to wait for a long time before I received health treatment. |  |  |  |  |  |  |
| 1. I could not pay for my medical care. |  |  |  |  |  |  |
| 1. Because of language barriers, I could not communicate with others. |  |  |  |  |  |  |
| 1. Because I do not know enough English, it has been difficult for me to deal with day to day situations. |  |  |  |  |  |  |
| 1. Because I do not know enough English, it has been difficult for me to interact with others. |  |  |  |  |  |  |
| 1. My family was exposed to poverty in my home country. |  |  |  |  |  |  |
| 1. I did not get good health care in my home country. |  |  |  |  |  |  |
| 1. I was forced to leave my home country due to poverty. |  |  |  |  |  |  |
| 1. My personal goals have been in conflict with family goals. |  |  |  |  |  |  |
| 1. Because of the lack of family unity, I have felt lonely and isolated. |  |  |  |  |  |  |
| 1. I had serious arguments with family members. |  |  |  |  |  |  |
